# Erysipelothrix rhusiopathiae Prosthetic Joint Infection of the Knee in an Immunocompromised Patient

**DOI:** 10.7759/cureus.108437

**Published:** 2026-05-07

**Authors:** Minu C Abraham, Asmita Gupte, Alaina S Ritter, Amy Vittor

**Affiliations:** 1 Division of Infectious Diseases and Global Medicine, University of Florida, Gainesville, USA; 2 Division of Infectious Diseases, Malcom Randall Veterans Affairs Medical Center, Gainesville, USA

**Keywords:** bone and joint infections, erysipelothrix rhusiopathiae, infectious diseases, orthopaedics, prosthetic joint infection

## Abstract

A male in his 70s with a remote history of a left total knee arthroplasty presented with acute left knee pain and swelling. He was a farmer with significant exposure to domestic and feral swine. CT of the knee showed a large complex left knee effusion and knee aspiration was concerning for infection. He underwent a debridement, antibiotics, and implant retention (DAIR) surgical procedure. Synovial fluid and operative cultures ultimately yielded* Erysipelothrix rhusiopathiae*, likely acquired from his animal exposures. He completed six weeks of intravenous daptomycin, followed by oral cefadroxil to complete a total of six months of antimicrobial therapy from the time of surgery. He had an excellent functional outcome with no recurrent infection at two years.

## Introduction

*Erysipelothrix rhusiopathiae* is a thin, facultatively anaerobic, Gram-positive rod that was first identified in the late 19th century in animals and humans [1-4]. It is a common commensal as well as a pathogen in many domesticated and wild animal species. In humans, *Erysipelothrix* infections are often associated with occupational and recreational animal exposure. The pathogen’s route of entry into the body is typically through contamination of a prior open wound or direct inoculation, such as a skin puncture [2]. Clinical manifestations of infection in humans include local skin infections (erysipeloid), diffuse cutaneous disease, osteomyelitis, joint infections, bacteremia, and endocarditis of native and prosthetic valves [2,3,5-7]. Prosthetic joint infections (PJIs) caused by *E. rhusiopathiae* have not been commonly reported in the literature. We describe a case of an *E. rhusiopathiae* left knee PJI that further illustrates the pathogenic potential of this organism in humans.

## Case presentation

A farmer in his 70s presented to our tertiary hospital with left knee pain and swelling for one week. His past medical history was significant for metastatic carcinoid tumor of the small bowel, hypertension, benign prostatic hyperplasia, colonic adenocarcinoma requiring right hemicolectomy two years prior to presentation, and a prior left total knee arthroplasty done nine years prior to presentation. His carcinoid tumor was initially diagnosed 12 years prior to presentation, and he was maintained on monthly lanreotide injections. The patient first noticed knee pain after lifting heavy equipment, which he initially attributed to overexertion. He denied any trauma to the knee. He subsequently developed worsening pain and swelling of the knee with associated chills, night sweats, general malaise, and lack of appetite, which prompted presentation for medical care.

Upon further discussion of his occupational history and exposures, the patient reported that he lived on a farm in rural Florida that had pigs, cattle, donkeys, and chickens. He reported spending significant amounts of time at a meat processing plant, where he hand-cut pig bones to feed to his farm dogs. He frequently sustained cuts and scrapes during this process, as well as while caring for the livestock, clearing his land, and gardening. He also reported hunting and eating wild boar. He had not travelled outside Florida recently.

In the emergency department, he was afebrile and hemodynamically stable, with a temperature of 37.7°C, blood pressure of 141/75 mmHg, and heart rate of 69 beats per minute. He appeared diaphoretic on exam and reported his knee pain was 7 out of 10. On examination, his left knee joint was mildly swollen with limited range of motion due to pain. Mild warmth was present, but there was no erythema. Laboratory findings were notable for elevated inflammatory markers and a normal white blood cell count, as noted in Table 1.

**Table 1:** Lab values. WBC: white blood cells; ESR: erythrocyte sedimentation rate; CRP: C-reactive protein; k/cmm: thousands per cubic millimeter; mm/hr: millimeters per hour; mg/dL: milligrams per deciliter.

Test	Result	Reference range
WBC	8.65 k/cmm	4.6-10.8 k/cmm
ESR	62 mm/hr	0-15 mm/hr
CRP	18 mg/dL	<0.3-0.5 mg/dL

**Figure 1:**
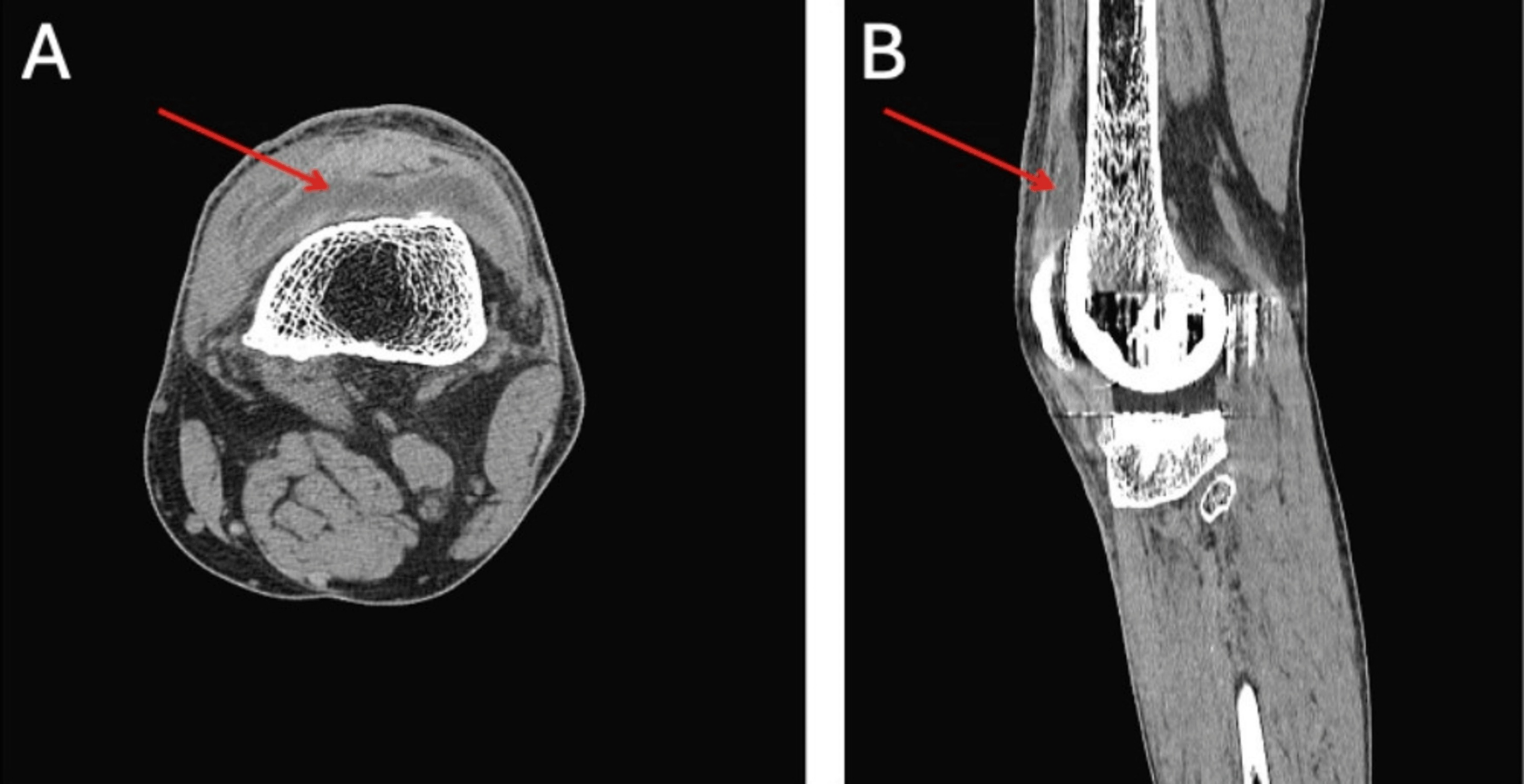
CT scan of the left knee. A: Axial view. B: Sagittal view. CT scan of the left knee demonstrating a large complex effusion with density higher than simple fluid (arrows), concerning for infection versus hemorrhage.

X-ray of the left knee showed normal alignment of his total knee arthroplasty. A computed tomography (CT) scan was subsequently obtained for more detailed characterization, which demonstrated a large complex left knee effusion concerning for infection versus hemorrhage (Figure 1).

Orthopedics evaluated the patient on the day of admission, given a high concern for a PJI. A left knee aspiration was performed, which was consistent with infection (Table 2).

**Table 2:** Results of synovial fluid analysis. cmm: cubic millimeter.

Synovial fluid parameter	Result	Reference range
Color	Yellow	Not specified
Appearance	Turbid	Not specified
Volume	3 mL	Not specified
White blood cells	44,990 per cmm	<200 per cmm
Red blood cells	8,000 per cmm	<1000 per cmm
Total neutrophil count	45,700 per cmm	Not specified
Granulocyte count	42,290 per cmm	Not specified
Lymphocyte count	1,799 per cmm	Not specified
Granulocyte percentage	94%	Not specified
Lymphocyte percentage	4%	Not specified
Mononuclear cell percentage	2%	Not specified
Crystals	None seen	None

Synovial fluid was sent for culture, and next-generation sequencing (NGS; MicroGenDX, Lubbock, TX) was also sent, given concern for a possible atypical organism that could be challenging to grow in culture. Blood cultures were obtained to rule out concomitant bacteremia. Empiric broad-spectrum intravenous antibiotic therapy with vancomycin and cefepime was initiated while awaiting culture results. An infectious disease consult was placed for management assistance.

Five days after presentation, the patient underwent left knee revision arthroplasty with incision and drainage, complete synovectomy, and tibial polyethylene exchange (also known as debridement, antibiotics, and implant retention, or DAIR). Intraoperatively, turbid, straw-colored synovial fluid and hyperemic synovium without frank purulence or necrosis were encountered. Samples of the synovium and scar tissue were sent for culture. The synovial fluid aspirate ultimately yielded a Gram-positive rod that could not be identified further at our institutional microbiology lab; pinpoint growth was first detected at two days. The sample was therefore sent to an external reference lab. Blood cultures remained negative, and a transthoracic echocardiogram did not show evidence of endocarditis.

Nine days after patient presentation, one out of three intraoperative cultures resulted in few *Erysipelothrix rhusiopathiae.* Susceptibilities were requested, but unfortunately could not be obtained due to insufficient growth of the organism in culture. Two weeks after presentation, NGS testing from the initial synovial fluid aspirate also returned as *E. rhusiopathiae*. This was consistent with the external reference lab report that finalized more than three weeks after presentation. Intraoperative fungal and acid-fast bacilli cultures were negative.

Based on the intraoperative culture results, the patient’s antibiotic regimen was transitioned to intravenous daptomycin, given intrinsic vancomycin resistance for *E. rhusiopathiae* reported in the literature [5,6,8-11]. The patient was discharged on hospital day 10. He completed six weeks of intravenous daptomycin, followed by oral cefadroxil suppression to complete a total of six months of antimicrobial therapy (including his initial IV course) from the time of surgery, as recommended per guidelines for a knee DAIR [12]. Of note, antibiotic dosing convenience, side effect profiles, and patient tolerance were considered when designing the final therapeutic regimen.

By the end of his antibiotic course, the patient had returned to working full-time on his farm with complete resolution of his left knee pain and normalization of his inflammatory markers. He reported chronic diarrhea as a result of his carcinoid tumor, but felt this may have worsened while on antibiotics. His gastrointestinal symptoms ultimately improved once antibiotics were stopped. He reported he was no longer cutting animal bones by hand, noted he would wear gloves when handling animal products in the future, and planned to exercise caution on the farm and while hunting to avoid injuries. At two years post surgery, he had no evidence of recurrent infection.

## Discussion

*E. rhusiopathiae* is a zoonotic pathogen with profound veterinary and economic impacts associated with a variety of animal species, including pigs, sheep, and turkeys [2]. Arctic animals used as a source of food, including muskoxen and seals, can also be affected [2,13]. In animals, *E. rhusiopathiae* causes a wide range of symptoms, including skin lesions, joint infection, bacteremia, and sepsis [14]. It can also survive in pickled and smoked meat and can be found in waste products and effluent from slaughterhouses [1,2,15]. In intensive pig farming areas, a significant percentage of the herd may be carriers, and the organism is spread through feces and nasal secretions [3,16]. Our patient likely developed transient bacteremia from cuts on his skin in the setting of livestock exposure, leading to PJI.

Exposure to infected animals can result in both localized and systemic *Erysipelothrix* infections in humans [2,3,5-7]. However, *E. rhusiopathiae* PJI is not commonly described. We conducted a literature review at the time of this case presentation and found eight prior reported PJI cases comprising three hip and five knee infections (Table 3).

**Table 3:** Literature review of prior Erysipelothrix rhusiopathiae PJI cases. DAIR: debridement, antibiotics, and implant retention; IV: intravenous; PO: oral; PJI: prosthetic joint infection.

Case	Joint​	Exposure	Surgery​	Sensitivity data​	Antibiotic therapy​	Recurrence-free follow-up
Traer et al. (​2008) [11]	Left knee	Tanning factory	2-stage revision​	Sensitive: Penicillin, flucloxacillin, gentamicin, erythromycin. Resistant: Vancomycin, rifampicin​	IV penicillin + PO levofloxacin, 3 weeks​; PO clindamycin + levofloxacin, 7 weeks​	1 year
Hocqueloux et al. (2010) [9]	Right knee	Pigs	Stage 1, then knee arthrodesis​	Not reported	IV imipenem + ofloxacin, 2 weeks​; PO clindamycin + ofloxacin, total 6 months​	32 months
Troelsen​ et al. (2010) [17]	Right hip	Hunting/hunting dog	2-stage revision​	Sensitive: Penicillin	IV penicillin, 3 weeks; PO amoxicillin, 2 months​	4 months
Gazeau et al. (2018) [4]	Left knee​	Unknown	1-stage revision. Recurrence: 2-stage​	Sensitive: Levofloxacin	After 1-stage revision: IV ceftriaxone, 3 months. After 2-stage revision: IV ceftriaxone + PO levofloxacin, 3 months​	2 years
Groeschel et al. (2019) [10]	Right knee​	Arctic animal meat	2-stage revision: declined stage 2​	Sensitive: Ampicillin, ciprofloxacin, erythromycin. Resistant: Vancomycin​	IV penicillin, 6 weeks; PO amoxicillin: 6 weeks​	1 year
Mahon​ et al. (2021) [8]	Right hip	Chicken manure	DAIR. Recurrence:​ 2-stage revision, declined stage 2​	Sensitive: Penicillin, erythromycin, cefotaxime, rifampicin, ciprofloxacin. Resistant: Vancomycin​	After DAIR: IV penicillin + gentamicin + metronidazole, 1 month; PO ciprofloxacin, indefinite​	18 months
Boukthir et al. (2022) [5]	Both knees; also bacteremia and endocarditis​	Farming, hunting, fishing	Bilateral knee DAIR. Left knee recurrence: ​2-stage​ revision	Sensitive: Amoxicillin	After DAIR: IV gentamicin (4 days) + IV amoxicillin, 6 weeks. First stage: IV amoxicillin, 3 months. Second stage: IV amoxicillin, 7 days​	2 years
McCall​ et al. (2023) [6]	Left hip; also​ bacteremia​	Wild boar	Partial revision (exchange of head, socket, and liner)​	Sensitive: Penicillin, ceftriaxone, clindamycin, ciprofloxacin, rifampicin. Resistant: Gentamicin, vancomycin	High dose IV penicillin, 2 weeks; PO ciprofloxacin, 6 weeks​	1 year
This report	Left knee	Domestic and feral swine	DAIR	Not reported	After DAIR: IV daptomycin, 6 weeks; PO cefadroxil, 6 months from surgery	2 years

Given the widespread distribution of this organism, additional undiagnosed or unreported cases are likely. Upon review of these eight published cases, a history of direct contact with animals was confirmed in seven cases (88%), which is consistent with the reported mode of transmission. Although all patients reported localized joint symptoms, only three (38%) had systemic symptoms such as rigors and fever, and two (25%) were ultimately found to have bacteremia. Upon review of lab abnormalities, an elevated CRP was found in seven (88%), and leukocytosis was found in two (25%). The culture positivity rate was 50% in arthrocentesis synovial fluid cultures, 100% in intraoperative cultures, and 25% in blood cultures.

All reported cases underwent surgical intervention in combination with antimicrobial therapy. Of the three patients who underwent either a one-stage revision or DAIR, all of them later required a two-stage revision in at least one joint due to recurrence of infection. In contrast, the five patients who underwent at least partial hardware removal did not experience recurrent infection. In this case report, our patient underwent DAIR, followed by six weeks of intravenous antibiotics and subsequent oral suppressive therapy to complete six total months of antibiotic therapy. Since his culture results did not return until after his surgery was complete, his surgical plan was designed empirically. At two years post surgery, he remained free of infection, with an excellent functional outcome. Therefore, this report demonstrates the potential for a successful clinical outcome after DAIR for *E. rhusiopathiae* PJI of the knee.

Medical management of *E. rhusiopathiae* can be challenging in part due to difficulties related to laboratory identification of this organism and its intrinsic resistance to vancomycin, a widely used empiric antibiotic for Gram-positive bacteria. It often grows slowly in the lab and yields small colonies [3,9]. On Gram stain, it can sometimes be mistaken for a Gram-negative organism as it decolorizes rapidly [1,2,18]. Characteristics that can aid in diagnosis include alpha hemolysis when grown in blood agar [2,18]. It also lacks beta hemolysis, is negative for oxidase, catalase, and indoles, is non-motile and non-spore-forming, and most strains produce hydrogen sulfide [2,3]. It can be identified using MALDI-TOF (matrix-assisted laser desorption/time-of-flight). Commercially available NGS tests that detect bacterial DNA in blood, other body fluids, and tissue may also be helpful in situations where growth in culture is challenging [18]. Typically, *E. rhusiopathiae* is sensitive to penicillins, fluoroquinolones, cephalosporins, clindamycin, and erythromycin and resistant to vancomycin and aminoglycosides [2-6,8,10,11,17].

## Conclusions

In conclusion, while *E. rhusiopathiae* is typically a zoonotic pathogen and commensal organism, it can also cause significant infection in humans. Diagnostic delays may occur given challenges in identifying this organism, and its inherent resistance to vancomycin can complicate medical management. Clinicians should consider this pathogen if Gram-positive rods are identified in a patient with PJI and animal exposures. Our patient remained infection-free two years after undergoing DAIR, indicating that for this particular case, this surgical approach combined with targeted antibiotic therapy effectively treated his *E. rhusiopathiae* knee PJI.
